# Wholegrain Intake and Risk of Type 2 Diabetes: Evidence from Epidemiological and Intervention Studies

**DOI:** 10.3390/nu10091288

**Published:** 2018-09-12

**Authors:** Giuseppe Della Pepa, Claudia Vetrani, Marilena Vitale, Gabriele Riccardi

**Affiliations:** Department of Clinical Medicine and Surgery, “Federico II” University, 80131 Naples, Italy; giuseppe.dellapepa@unina.it (G.D.P.); c.vetrani@libero.it (C.V.); marilena.vitale@yahoo.it (M.V.)

**Keywords:** wholegrain, diabetes diet, type 2 diabetes mellitus, plasma glucose, plasma insulin, diabetes prevention

## Abstract

Type 2 diabetes mellitus (T2DM) is one of the most common metabolic diseases and represents a leading cause of morbidity and mortality because of its related complications. The alarming rise in T2DM prevalence worldwide poses enormous challenges in relation to its social, economic, and a clinical burden requiring appropriate preventive strategies. Currently, lifestyle modifications—including approaches to promote a moderate body weight reduction and to increase regular physical exercise—are the first crucial intervention for T2DM prevention. In the light of the difficulty in reducing body weight and in long-term maintenance of weight loss, quality changes in dietary patterns—in terms of macro and micronutrient composition—can also strongly affect the development of T2DM. This may provide a more practical and suitable preventative approach than simply implementing caloric restriction. Along this line, there is increasing evidence that wholegrain consumption in substitution of refined grains is associated with a reduction of the incidence of several non-communicable chronic diseases. The aim of the present review is to summarize the current evidence from observational and randomized controlled clinical trials on the benefits of wholegrain on T2DM prevention and treatment. Plausible mechanisms by which wholegrain could act on glucose homeostasis and T2DM prevention are also evaluated. Altogether, the totality of the available evidence supports present dietary recommendations promoting wholegrain foods for the prevention and treatment of T2DM.

## 1. Introduction

Type 2 diabetes mellitus (T2DM) is one of the most common metabolic diseases with 415 million cases estimated globally in 2015; this number is expected to increase dramatically in the next decades reaching 642 million by 2040 [1]. T2DM represents a leading cause of morbidity and mortality worldwide because of its related microvascular and macrovascular complications. The alarming rise in T2DM prevalence worldwide—including low-income countries and adolescents/young adults—as well as its heavy impact on longevity and quality of life, poses enormous challenges in terms of social, economic, and clinical aspects, urging appropriate preventive strategies [2]. The progressive diffusion of western dietary habits and low physical activity, and the strictly related global increase in overweight/obesity are the major determinants of the growth of T2DM prevalence observed in the last decades together with the increased longevity connected to the improvements of diabetes care [3]. In particular, the incidence of T2DM is very high in overweight/obese individuals with visceral adiposity and its linked pathological conditions characterized by interrelated alterations in metabolic and vascular functions such as hyperglycemia, dyslipidemia, insulin resistance, and hypertension [4].

Lifestyle interventions aiming at reducing body weight and increasing regular physical activity represent the cornerstone of T2DM prevention and management. Strong evidence from randomized controlled trials (RCTs) in at risk individuals from different populations supports the notion that lifestyle modifications—including a healthy diet, a 7% loss of initial body weight, and a moderate-intensity exercise consisting of at least 150 min/week—represents a very effective strategy for T2DM prevention and treatment [5].

Beside physical activity and body weight reduction, a healthy diet represents an additional tool to prevent and treat T2DM over and above its effects on body weight [5]. In this respect, it is important to underline that body weight reduction and its long-term maintenance are very difficult to be achieved in a large proportion of the at risk population. Therefore, changes in dietary habits, able to reduce the risk of T2DM [5] independently of calorie restriction, may represent an important resource in the context of a practical and suitable preventive approach at the population level. Along this line, a number of observational studies have highlighted food items that are associated with a reduced risk of T2DM: Fruits, nuts, fish, vegetables, non-tropical vegetable oils, wholegrains, beans, and yogurt [6]. Recently, a comprehensive meta-analysis has indicated that in addition to foods associated with a lower risk, there are also food items that are associated with an increased risk of T2DM: Red meat, processed meat, eggs, and sugar-sweetened beverages [7]. A dietary pattern based on the preference of vegetable products and on moderate consumption of animal foods resembles the traditional Mediterranean Diet that has shown to be able to reduce the incidence of T2DM by as much as 30%, despite the fact that body weight did not change [8].

Among all food items associated with the incidence of T2DM in observational studies, unambiguous evidence has emerged over the last few decades on the possible role of wholegrain consumption in preventing this disease as well as many other widely chronic non-communicable diseases [9].

Several definitions are proposed for “wholegrain” and all give special importance to the intact grain and its three components: Endosperm, germ, and bran [10], which should be present in the same relative ratio existing in the intact caryopsis. The definition proposed by the European HEALTHGRAIN Consortium also accepts small losses of kernel’s components—2% of the grain or 10% of the bran—that may occur through processing to preserve safety and the quality of the product [11]. A further distinction should be made to separate intact kernels (i.e., intact, un-milled wholegrains) from milled wholegrains (i.e., wholegrain flours and the products made from them). Wholegrains are represented by cereals (i.e., wheat, rice, maize, rye, oat, millet, barley, sorghum, teff, and triticale), pseudo-cereals (amaranth, buckwheat, and quinoa), and wild rice [12]. While wholegrains are characterized by the presence of all kernel constituents, great variability in terms of macronutrient/micronutrient composition and content of bioactive compounds has been described for the various cereal classes (Table 1).

Wholegrain foods include breads, breakfast cereals, pasta, biscuits, and grain-based snack foods [17]. In comparison to refined grains, they are rich in dietary fiber, resistant starch, antioxidants, and other important micronutrients such as folic acid and other vitamins [13,14,15,16,18]; altogether, these components of wholegrain have relevant functional properties that can at least, in part, justify its health benefits [19].

Thus, the aim of the present review is to summarize the available evidence derived not only from epidemiological studies, but also from intervention trials on the possible protective effects of wholegrain foods on T2DM prevention and treatment.

## 2. Methods

We have reviewed the evidence from observational studies, clinical trials, randomized clinical trials (RCTs), and meta-analyses published in the last fifteen years on Pubmed, which evaluated the relationship between wholegrain consumption and T2DM. We have focused our search on studies performed in humans in which the effect of individual wholegrains/wholegrain foods or wholegrain rich diets were compared with diets or foods based on refined grains with a similar energy intake and macronutrient composition.

We have first considered studies performed in free-living adults who were either healthy or with some known risk factors for T2DM. In observational studies, the incidence of T2DM represented the main outcome. Conversely, due to the absence of long-term RCTs on T2DM incidences, we have included, in our search, shorter term trials—with the exclusion of acute-meal studies—on surrogate endpoints known to be major established T2DM risk factors: Impaired glucose tolerance, plasma glucose, insulin resistance, overweight/obesity, and abdominal obesity. The second part of our search has been performed in patients with T2DM taking into consideration both observational studies and intervention trials. With respect to RCTs on the effects of wholegrain intake in T2DM patients, we have considered studies performed in individuals with clinically established T2DM; changes in fasting and postprandial plasma glucose and glycated hemoglobin (HbA1c) were the main outcomes evaluated in these studies. Finally, we have also evaluated plausible mechanisms by which wholegrain could act on glucose homeostasis and T2DM prevention.

## 3. Wholegrain Intake and T2DM Prevention

### 3.1. Epidemiological Studies

A regular intake of wholegrain has been consistently associated with a lower risk of T2DM in different populations, as shown by the three meta-analyses so far published [20,21,22]. Most of the studies included in these meta-analyses, particularly those performed in the USA, which used identical methods to classify breakfast cereals and other grain products, as whole or refined grain, to form whole and refined-grain food groups and to calculate the daily consumption of wholegrain and cereal fiber. The association between habitual wholegrain consumption and a lower incidence of T2DM remained significant after adjusting lifestyle factors, i.e., physical activity, BMI, waist to hip circumference, smoking, alcohol, energy intake, and education. Priebe et al. reported that 11 prospective studies consistently showed a reduced T2DM risk for a higher intake of wholegrain (between 27% and 30%) or cereal fiber (between 28% and 37%) [20]. The comprehensive meta-analysis of Ye et al. [21] showed that the overall estimated multivariable-adjusted relative risk of T2DM development by comparing the highest intake of wholegrain—an average of 48–80 g/day—with the lowest, which was reduced by 26% (RR = 0.74, 95% CI: 0.69, 0.80). It is important to underline that, although this meta-analysis reports the average wholegrain intake in relation to the reduced risk of T2DM development, the optimal intake of wholegrain for T2DM prevention could not be established because the shape of the dose-response relationship was not investigated. This was instead clarified in a dose-response meta-analysis of cohort studies conducted by Aune et al. [22], which showed that the maximal reduction of T2DM incidence (32%) was associated with 2–3 servings/day (60–90 g/day) of wholegrain (RR = 0.68, 95% CI: 0.58, 0.81), and no major further reductions were achieved with higher intakes. The association between habitual wholegrain intake and a lower rate of T2DM or impaired glucose regulation is confirmed by studies in which a biological marker of wholegrain wheat or rye intake (an alkylresorcinol metabolite) was utilized [23,24,25].

The meta-analysis by Aune et al. [22] also gives some information on the association between specific subtypes of wholegrain foods (bread, breakfast cereals, and brown rice) that were largely responsible for the association of wholegrain with a reduced T2DM development. However, the analyses on the relationship between specific wholegrain foods with T2DM incidence were based on few studies and need further confirmation. The lack of studies on specific foods or cereal types is mainly due to the different sources of wholegrain foods utilized in various populations [26]. In fact, the main source of wholegrain was bread in Scandinavian countries [27], bread and breakfast cereals in the USA [28], brown rice, unrefined maize, and sorghum in some African countries [29], and brown rice in Asia [30]. At variance with the finding of a reduced incidence of T2DM in habitual wholegrain consumers, no association or even an increased risk of T2DM has been consistently reported for habitual consumers of higher amounts of refined grains [31,32,33].

In conclusion, epidemiological studies provide strong and consistent evidence on the association of habitual wholegrain consumption with a lower incidence of T2DM, supporting scientific recommendations from authoritative bodies to consume at least two-to-three servings/day of wholegrain foods with the expectation to contribute to the prevention of T2DM.

### 3.2. Intervention Trials

There are no randomized controlled clinical trials assessing the incidence of T2DM in relation to wholegrain intake; this is obviously due to the complexity of the design of such a study needing both a long duration of the intervention and a large sample size.

## 4. Wholegrain Intake and T2DM Risk Factors

### 4.1. Body Weight/Body Fat

Overweight/obesity are powerful modifiable risk factors for T2DM. Observational evidence has consistently shown that a mean consumption of two-to-three servings/day (30–45 g/day) of wholegrain is associated with a lower body mass index (BMI) and a decreased body weight gain over time [34,35,36,37]. However, results obtained in RCTs are less convincing (Table 2).

In fact, a meta-analysis of RCTs has shown that habitual wholegrain intake does not induce any significant reduction of both body weight or waist circumference in overweight people [38]. Data from 2060 participants were included in the analysis. While in this paper wholegrain intake did not show any beneficial impact on body weight (weighted difference: 0.06 kg; 95% CI: −0.09, 0.20 kg; *p* = 0.45), conversely, it induced a small effect on the amount of body fat (weighted difference: −0.48%; 95% CI: −0.95%, and −0.01%; *p* = 0.04) in comparison to a control diet based on refined cereals. The lack of major effects on body weight of wholegrain has been confirmed by a more recent RCT not included in the meta-analysis; this trial compared wholegrain (90 g/day) obtained from different types of cereals (57% wheat, 21% rice, and 16% oat) with a similar combination of refined grains during a six-week intervention [39]. Substantial reductions in body weight, fat mass, systolic blood pressure, total cholesterol, and low-density-lipoprotein (LDL) cholesterol were observed during both diet periods, with no significant difference between the wholegrain and the refined cereal diet. However, there was an improvement in diastolic blood pressure three times greater in overweight and obese adults when they consumed wholegrain, as compared with a refined-grain diet.

Interestingly, more recent RCTs have focused on the effects of specific types of wholegrain cereals (rye, wheat and oat) on anthropometric parameters. Seventy overweight/obese adults participated in a six-week randomized parallel study in which they replaced their habitual cereal foods with refined wheat, wholegrain wheat or wholegrain rye within an ad-libitum diet. The wholegrain rye-based diet significantly reduced body weight by roughly one kg, as compared to the refined wheat-based diet. Conversely, no effect on body weight was observed after the diet based on wholegrain wheat. While it was an ad-libitum intervention, no difference in total energy intake was observed between the three diets; however, the energy intake from study products was ~200 kcal lower in the wholegrain rye group when compared with that in the refined wheat group (*p* < 0.05). This suggests that the observed effects of wholegrain rye on body weight may be, at least in part, mediated by an increased satiation as indicated by the reduction in energy intake from wholegrain rye foods without compensation from other components of the diets [40]. A beneficial effect on body weight regulation has been shown for the regular consumption of wholegrain oat-based foods, but in this study, participants were people with diabetes [41].

In order to interpret the epidemiologic associations between wholegrain consumption and reduced body weight and adiposity, possible effects of wholegrain on energy metabolism should also be considered. In fact, it has been recently demonstrated that substituting wholegrains (mostly wheat, but oat and brown rice were also included) for refined grains in a 6-week randomized trial favorably affects energy-balance in healthy men and postmenopausal women; this dietary maneuver increased the resting metabolic rate as well as stool energy excretion [42].

In summary, the available evidence, mainly based on observational studies, indicates that a regular consumption of wholegrain is associated with a lower BMI and a decreased body weight gain over time. These findings are not always consistent with results of intervention trials, which overall indicate that wholegrain might have a small beneficial effect on body fat reduction, while it has no measurable impact on body weight for overweight people. The effects of wholegrain foods on body weight regulation may be more important for specific cereal types, namely oats and rye [40,41].

The inconsistency of the findings from observational and intervention studies may partly rely on the difficulty to reproduce, in experimental conditions, the long and complex natural history of overweight. In this context, intervention studies on weight reduction are not necessarily relevant in relation to the development of overweight.

### 4.2. Metabolic Syndrome/Insulin Resistance

The metabolic syndrome describes a cluster of abnormalities that are associated with an increased risk of T2DM. Set-aside the diagnostic criteria established by the NCEP-ATPIII in 2003 [43]—waist circumference, plasma glucose, plasma triglycerides, high-density-lipoprotein (HDL) cholesterol, and blood pressure—a core component of the metabolic syndrome is impaired insulin sensitivity. In the last years, several cohort studies have shown an association between habitual wholegrain intake and a reduced risk of the metabolic syndrome [44,45,46,47].

As for the association between wholegrain intake and insulin resistance, to the best of our knowledge, only cross-sectional studies have focused on this topic. The results of a cross-sectional study performed in the USA have clearly indicated that a higher habitual intake of wholegrain (dark bread, high-fiber, and cooked cereals) is associated with a better insulin sensitivity [48]. Additionally, in a well-characterized population of Danish school children, intakes of wholegrains (rye, wheat, and oat) were inversely associated with serum insulin. Among the various types of wholegrain cereals, oats showed the strongest association with lower serum insulin values [49].

Despite the fact that several RCTs have explored the impact of wholegrain on insulin sensitivity [50,51,52,53,54,55,56,57,58,59], a meta-analysis of the available RCTs on this topic has never been performed. The only exception is the meta-analysis of Marventano et al. [60], which only includes studies in which insulin sensitivity was evaluated by the measurement of fasting insulin concentrations and/or the homeostatic model assessment-insulin resistance (HOMA-IR) index. The results of the meta-analysis showed no evidence of an effect on HOMA-IR in medium-term interventions comparing wholegrain consumption with a refined cereal diet (MD = −0.18, 95% CI: −0.48, 0.13). Results of the available RCTs on insulin sensitivity, evaluated by any possible type of measurement, are reported in Table 3.

Overall, the available evidence is inconsistent, since in some studies habitual wholegrain consumption was effective in improving insulin sensitivity [50,58]; while in most other studies, there was no effect.

The inconclusive results may be due to the huge variability in the methodology employed in these RCTs; this refers particularly to the cereals used in the trials, the duration of the exposure, and particularly, the methodology to assess insulin resistance/sensitivity. A tentative interpretation may be proposed to try to reconcile the evidence from observational studies and intervention trials on insulin sensitivity/metabolic syndrome (as well as observational studies on the incidence of T2DM that is pathophysiologically linked to impaired insulin sensitivity):

(1) The metabolic impact of wholegrain is mostly confined to the postprandial period; this has been clearly shown by an intervention trial from our group [61] that compared a diet based on wholegrain cereal foods with a refined cereal diet of identical nutrient composition. The wholegrain diet significantly reduced the postprandial insulin response by as much as 30%, in comparison with the control diet, despite similar postprandial glucose levels. In the same study, fasting insulin sensitivity was evaluated by both the HOMA index and the insulin sensitivity index measured during an Intravenous Glucose Tolerance Test was not at all influenced by the wholegrain intake [57]. The impact of wholegrain, specifically on postprandial insulin metabolism, has been recently confirmed in obese people at risk of T2DM [58]. Further support to the importance of the postprandial metabolism, in evaluating the impact of wholegrain on insulin sensitivity, comes from a study in which objective markers of wholegrain wheat or rye intake were employed and insulin sensitivity was evaluated after an oral glucose challenge. In this study, the alkylresorcinol C17:0/C21:0 ratio measured in non-fasting conditions was positively associated with insulin sensitivity indices measured after the oral glucose load—Matsuda ISI (*p* = 0.026) and disposition index (*p* = 0.022)—in a pooled analysis of the wholegrain and the control diet groups at the end of the intervention, and after adjustment for confounders [62].
(2) Study duration has to be long enough to allow stable changes in the intestinal ecosystem that may eventually optimize the fermentation of fiber from wholegrain cereals.
(3) The metabolic benefits may be more relevant for cereal types that provide larger amounts of indigestible carbohydrates, and particularly, types of dietary fiber that are more fermentable in the gut (i.e., oat, barley) [63].

### 4.3. Blood Glucose Regulation

Epidemiological cohort studies support the important role of blood glucose levels in the fasting state, and even more after an oral glucose challenge, as they are predictors of future cardiovascular events. A relationship between wholegrain intake and lower plasma glucose levels has been reported in cross sectional studies [45,47].

Unfortunately, there is only one epidemiological study evaluating the association between habitual wholegrain consumption and changes of plasma glucose levels over time, and it demonstrates that habitual wholegrain consumption (mostly rye bread but also other cereals) is associated with a reduced rate of deterioration of glucose tolerance from normal to impaired [64].

As for the evidence from the RCTs [61,65] (Table 4), meta-analyses [60] carried out in healthy subjects have shown no effects of wholegrain consumption on fasting glucose and insulin concentrations.

In relation to the blood glucose regulation in the postprandial period, evidence from meal studies, employing in most cases wholegrain oat, barley, or rye, indicates that both postprandial glucose and insulin responses are lowered in healthy people when these cereals are consumed as wholegrains rather than in their refined form. However, the results of the acute studies, included in this meta-analysis, cannot be extended to all wholegrain cereals, and in particular, to wholegrain wheat that is more widely utilized worldwide [60]. In this paper, a meta-analysis was also performed on medium term RCTs that compared wholegrain rich diets with diets based on refined grains (Table 4). Unfortunately, these studies are rather few, and overall, the meta-analysis did not show significant differences in fasting plasma glucose levels with wholegrain diets, as compared to the refined cereal ones.

However, looking at the very few medium term RCTs in which wholegrain products were based on oat or barley, a clear improvement on post-prandial glucose response could be depicted. A meta-analysis on the effects on glucose metabolism of oat-based foods, in comparison to other cereal foods, has shown a clear reduction of the postprandial plasma glucose response [63].

In synthesis, the evidence on the relationship between habitual wholegrain consumption and plasma glucose levels, both in the fasting state and in the postprandial period, is limited and does not allow the drawing of any definite conclusions. The only exceptions are wholegrain foods based on oat and barley, for which the evidence of a beneficial impact on postprandial plasma glucose values is rather convincing. This is in line with the health claim approved by the European Food Safety Authority (EFSA), which states that: “consumption of beta-glucans from oat or barley contributes to the reduction of the glucose rise after a meal” [66]. Indeed, the highest concentration of β-glucan is found in barley and oat [67].

## 5. Effects of Wholegrain Consumption in Patients with T2DM

### 5.1. Observational Studies

There are no data on the relationship between habitual wholegrain consumption and markers of blood glucose control in epidemiological studies performed in T2DM.

### 5.2. Intervention Trials 

Blood glucose control is the most important target, for diabetes management, in order to prevent its micro and macro-vascular complications.

Few studies focusing on wholegrain/wholegrain foods and their effects on blood glucose control in T2DM patients are available. One trial evaluated a diet based on bread and breakfast cereals high in fiber (19 g/day additional cereal fiber) for three months in a group of T2DM patients, in comparison with a control diet that was low in cereal fiber. This study demonstrated that the diet based on high-fiber cereal foods did not improve conventional markers of glycemic control or risk factors for coronary heart disease [68]. A systematic review with a meta-analysis of RCTs has recently summarized the effects of a high fiber diet on glycemic control in people with T2DM, showing that increasing fiber intake, in particular soluble fiber, significantly improves the glycemic control in these patients. Obviously, wholegrain foods give an important contribution to a higher intake of dietary fiber, but in this meta-analysis, the specific role of wholegrain foods was not evaluated [69].

More recently, studies [41,70,71] have been performed focusing specifically on some cereal types (Table 5).

A meta-analysis of 14 RCTs and 2 uncontrolled observational studies [70] show that in T2DM patients, regular oat intake (50–100 g/day for 1–4 weeks) induces a significant reduction of HbA1c (−0.42%) and fasting plasma glucose levels (−0.39 mmol/L), in comparison with a control diet based on other cereals or other carbohydrate foods. Furthermore, oatmeal significantly reduced the acute postprandial glucose and insulin responses, compared with a control meal [70]. Similarly, Shen et al. [71] reported that T2DM patients who were given oat-based products from 2.5 to 3.5 g/day for 3 to 8 weeks had significantly lower HbA1c values (−0.21%) and fasting plasma glucose levels (−0.52 mmol/L), in comparison with those on a control diet.

Li et al. [41] reported that diabetic patients randomly allocated to eat a diet containing 100 g oat/day or a control diet for one month had a significant reduction of post prandial plasma glucose levels (−1.48 mmol/L; 95% CI: −2.57, −0.39), together with a significant improvement of insulin sensitivity (HOMA-IR = −1.77 mU·mol/L^2^; 95% CI: −3.49, −0.05) when they were eating the oat-based diet; in addition, with this diet, both total (−0.33 mmol/L; 95% CI: −0.56, −0.10) and LDL cholesterol (−0.22 mmol/L; 95% CI: −0.41, −0.03) were significantly reduced. In the one-year follow-up, significant benefits on weight reduction (−0.89 kg; 95% CI: −1.56, −0.22) and HbA1c (MD: −0.64%; 95% CI: −1.19, −0.09) were still manifest in the group that was assigned to wholegrain oat.

In conclusion, considering the totality of the available evidence, the scientific support for the beneficial role of wholegrain on the metabolic control of T2DM patients is scanty. However, the available literature on this topic includes only few studies, and most of them present significant methodological limitations, including a small sample size and a relatively short follow-up. Focusing on specific types of wholegrain cereals, meta-analyses of RCTs clearly indicate that wholegrain oat consumption is able to improve blood glucose control in T2DM patients.

Other studies are needed in order to clarify whether substituting wholegrain for refined cereal products induces measurable health benefits in T2DM patients, in the long term, in relation to the metabolic control and the risk of chronic complications, i.e., cardiovascular diseases.

## 6. Plausible Mechanisms by Which Wholegrains Might Protect against T2DM

**Figure 1:**
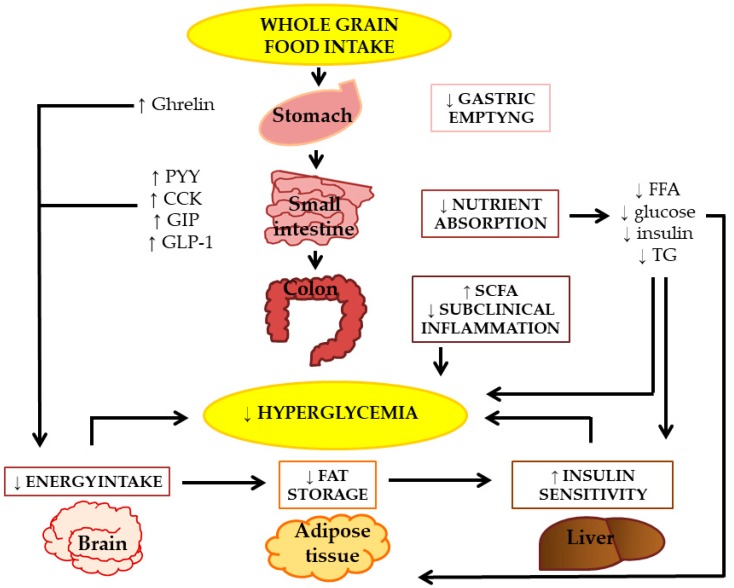
Schematic outline of plausible mechanisms of action by which wholegrain could influence glucose homeostasis and T2DM risk development. PYY: peptide YY; CCK: cholecystokinin; GIP: gastric inhibitory peptide; GLP-1: glucagon like peptide 1; FFA. Free fatty acids; TG triglycerides; and SCFA: short chain fatty acids, ↓ decrease, ↑ increase.

Many components of wholegrain can play a role in improving glucose metabolism, thus contributing to T2DM prevention (Figure 1). Among them, dietary fiber has been extensively investigated since wholegrain foods are a good source of fiber (ranging from 9 to 17 grams for every 100 grams of an edible portion). Fibers from wholegrain cereals are mostly of the insoluble type—i.e., cellulose, hemi-celluloses, and lignin—with the exception of barley and oats that are relevant sources of soluble fiber, i.e., beta-glucan, pentoses, and arabinoxylan [72]. In particular, the concentration of beta-glucan varies from 0.1% dry weight in corn, to 4.1% in barley, while arabinoxylan ranges from 4.7% in corn to 9.7% in oats; the concentration of cellulose varies from 1.4% dry weight in rye, to 8.2% in oats, while lignin ranges from 1.1% in corn, and up to 6.6% in oats [67].

Many plausible mechanisms could be involved in the relationship between wholegrain fiber and improvements of glucose homeostasis. First, it could play a significant role in body weight regulation [37]. In fact, fiber contributes to lower the energy density of wholegrain foods, as compared to the refined ones [73]; furthermore, the larger size of starch granules in wholegrain foods and their structural integrity require a higher chewing rate that is strictly related to the oro-sensory stimulation and to satiation, possibly contributing to a reduced food intake [74,75]. Second, fiber (particularly the viscous type) is able to increase gastric distension contributing to stimulate satiety signals and to increase hormones involved in body weight regulation and energy homeostasis, as well as in glucose control [76,77,78,79]. In fact, in a recent study from our group [79] in which wholegrain pasta was compared with a regular one, the desire to eat and the sensation of hunger were lower after the wholegrain pasta (−16%, *p* = 0.04, and −23%, *p* = 0.004, respectively); in addition, satiety was higher (+13%; *p* = 0.08) compared with the control pasta. Changes in appetite ratings correlated with polypeptide YY (PYY) plasma levels (*p* < 0.03). However, wholegrain pasta did not influence the overall energy balance. Similarly, increments of gut hormones and of the insulin sensitivity index were observed following a three-day intervention with a barley kernel-based product [80].

The influence of dietary fiber on glucose metabolism has been attributed both to soluble (viscous) and insoluble fibers. Viscous fiber physiologically modulates the postprandial glycaemic response by delaying gastric emptying and small bowel transit time; this can stimulate secretion of intestinal hormones involved in glucose metabolism. In fact, both of them decrease starch accessibility to α-amylase and reduce glucose diffusion through the unstirred layer [81]. This mechanism is strengthened when the cereal grains are not milled. Unfortunately, few wholegrain cereal foods available for consumers and utilized in intervention studies are based on intact kernels; food structure, besides other features of wholegrain cereal products, has a strong impact on postprandial metabolism by modulating the rate of nutrient digestion (accessibility to digestive enzymes) and absorption in the small intestine. Furthermore, viscous fiber reduces the postprandial glycaemic response by delaying gastric emptying and small bowel transit time; this can stimulate the secretion of intestinal hormones involved in glucose metabolism.

Interestingly, the effects on the postprandial glycemic response and on satiety of isolated cereal fibers have been compared with those obtained with intact kernel wholegrain products in acute meal studies [82,83]. The outcomes of these studies indicates that both a wheat bread enriched with oat beta glucan (highly viscous) and an intact kernel rye bread similarly reduce the postprandial blood glucose response, as compared with a refined wheat bread. Conversely, a wheat bread enriched with wheat arabinoxylan (less viscous) had no major impact on the postprandial blood glucose response. However, both beta glucan and arabinoxylan increased satiety feelings in comparison with refined wheat bread; the magnitude of their effects is similar to that of intact rye kernel.

Wholegrain fiber—as well as dietary fibers from other sources—can be fermented by the intestinal microbiota with several beneficial metabolic effects. In fact, short chain fatty acids (acetate, propionate and butyrate) represent the main products of fiber fermentation and they have been shown to influence glucose metabolism by reducing plasma levels of non-esterified fatty acids, that impair insulin sensitivity, and by increasing hepatic glycolysis and decreasing hepatic glucose production, thus lowering plasma glucose levels [37,84]. Indeed, in a study from our group in subjects with the metabolic syndrome, higher plasma propionate levels were associated with a better insulin sensitivity after a 12-week of a wholegrain-based dietary intervention [85].

In addition, wholegrain consumption has been shown to influence the composition of the gut microbiota; this is now recognized as a major determinant of the interplay between diet and metabolic health. In particular, in some studies, the chronic intake of wholegrain wheat [86], rye [65], and barley [87] induced a decrease of colonic bacterial species that produce molecules able to trigger endotoxemia (i.e., lipopolysaccharides and peptidoglycans) and to promote chronic low-grade inflammation (by tumor necrosis factor-α) and insulin resistance.

Wholegrain can influence glucose control and T2DM risk by multiple mechanisms of action. The lower energy density of wholegrain foods could play a significant role in reducing the energy intake; this is, furthermore, enabled by the larger size of starch granules in wholegrain foods and by their structural integrity, which requires a higher chewing rate, thus increasing satiation. Fiber from wholegrain is able to increase gastric distension and to delay intestinal transit time contributing to stimulate satiety signals and to increase hormones (Ghrelin, PYY, CCK, GIP, GLP-1) involved in energy homeostasis and plasma glucose control. A lower energy intake leads to a decrease of body fat with an improvement of insulin sensitivity. Furthermore, fiber from wholegrain delays nutrient absorption (glucose, FFA) at the intestinal level, and this reduces the insulin demand and stimulates fat oxidation, thus contributing to reduce fat storage. In the colon, wholegrain modulates the composition of gut microbiota and promotes fiber fermentation with production of SCFA. This improves insulin sensitivity at the liver site and reduces subclinical inflammation. A long term improvement of plasma glucose level can be the consequence of lower fat storage, improved insulin sensitivity at the liver site together with a reduced subclinical inflammation and a reduced energy and nutrient intake. Finally, bioactive compounds present in wholegrains (i.e., phenolic compounds, phytosterols, betaine, and carotenoids) may contribute to improve insulin sensitivity and reduce the development and progression of T2DM by acting on the oxidative stress, the transcription of inflammatory cytokines, and subclinical inflammation.

A possible beneficial role has been ascribed to some bioactive compounds present in wholegrain [72,88]. In particular, phenolic compounds, phytosterols, betaine, and carotenoids, for their antioxidant and anti-inflammatory properties, may contribute to reduce the development and progression of T2DM by hampering the oxidative stress, the transcription of inflammatory cytokines and chronic low-grade inflammation [14,89], thus improving insulin sensitivity [90]. Our group has recently shown that a diet based on natural products rich in polyphenols improves glucose tolerance and insulin sensitivity in non-diabetic people and lowers the postprandial triglyceride response [91].

Finally, wholegrain is a good source of vitamins and minerals that may also play a role in glucose metabolism. The most representative vitamins in wholegrain are the B complex vitamins, ranging from one mg (for thiamin) to 11 mg (for nicotinic acid) in every 100 g of wholegrain; vitamin E is also present in a good quantity (two-to-seven mg/100 g wholegrain). Vitamin B complex may contribute to the regulation of hepatic glucose uptake [92], while vitamin E may be beneficial in reducing the oxidative stress and chronic low-grade inflammation associated with obesity, metabolic syndrome, and insulin resistance [93,94,95]. With respect to minerals, wholegrain is a good source of iron, magnesium, zinc, manganese, and selenium [72]. Magnesium, in particular, has been suggested in some studies to contribute to the regulation of insulin-mediated glucose uptake, and more generally, to the improvement of insulin sensitivity [96]. Zinc may support the signal transduction of insulin and could improve glucose homeostasis by reducing the production of some cytokines and oxidative stress involved in β-cell death [97]. Obviously, many of these mechanisms are operative in people with severe deficits of these micronutrients. Further studies in humans are needed to highlight their metabolic relevance in general populations, or at least in people with less severe deficits like elderly people.

## 7. Conclusions

Findings from large observational-prospective or cross-sectional studies reviewed in this paper have consistently demonstrated that a higher intake of wholegrain is associated with a lower risk of T2DM, as well as an improvement of its major risk factors, i.e., overweight/obesity, plasma glucose regulation, postprandial hyperinsulinemia, and insulin resistance. Moreover, habitual wholegrain consumption is also associated with a reduced risk of other chronic diseases and with a better nutritional quality of the diet, due to greater intakes of micronutrients [17,98]. With respect to T2DM, epidemiological evidence indicates that individuals who consume an average of two-to-three daily servings (60–90 g/day) of wholegrain have a 21–32% reduction in the incidence of T2DM compared with those who rarely or never consume wholegrain. This amount can be easily achieved by substituting at least half of the refined cereal foods in the habitual diet with the wholegrain ones.

Many intervention trials have been undertaken in order to investigate whether wholegrain consumption is able to improve major risk factors for T2DM; however, findings from these studies have not been as impressive as those from the observational ones. So far, the evidence from these trials do not allow us to draw definite conclusions about the preventive efficacy of wholegrain foods on the development of T2DM or its major risk factors. This represents a remarkable research gap that needs to be filled by well-designed, adequately powered, and randomized clinical trials with sufficient duration to be able to ascertain the long-term effects of wholegrains on T2DM prevention and treatment.

However, given the strength and the reproducibility of the evidence related to the possible benefits of wholegrain for prevention of T2DM achieved in epidemiological studies—as well as the consistency of the observational data showing other better health outcomes associated with habitual wholegrain consumption—it seems wise to include wholegrain foods in the dietary recommendations for T2DM prevention and treatment. This also seems appropriate in view of the lack of relevant adverse effects associated with wholegrain intake. Special emphasis should be given to specific types of wholegrain cereals, like oats and barley, for which a beneficial impact on glucose metabolism has been more clearly demonstrated in intervention studies. Regular consumption of wholegrain is now recommended by nutritional guidelines in many countries [17] and by statements from major scientific societies in the field of diabetes [5,99]. For the time being, on the basis of the evidence reviewed in this paper, two-to-three servings per day of wholegrain (60–90 g/day), as indicated by dietary recommendations for T2DM prevention and treatment [5], represents an appropriate and achievable goal for the general population, and even more for individuals at increased risk of T2DM.

## Figures and Tables

**Table 1:** Nutrient composition of the most widely consumed wholegrain cereals.

Nutrient	Reference	Wheat	Brown Rice	Maize	Oat	Rye	Barley
**Macronutrient** (g/100 g)	[13]						
Carbohydrate		71.2	76.2	74.3	66.3	75.9	73.4
Lipid		1.5	3.2	4.7	6.9	1.6	2.3
Protein		12.6	7.5	9.4	16.9	10.3	12.5
Fiber		12.2	3.6	7.3	10.6	15.1	17.3
**Minerals** (mg/100 g)	[13]						
Calcium		29	33	7	54	24	33
Iron		3.19	1.80	2.71	4.72	2.63	3.60
Magnesium		126	143	127	177	110	133
Phosphorus		288	264	210	523	332	264
Potassium		363	268	287	429	510	452
Sodium		2	4	35	2	2	12
Zinc		2.65	2.02	2.21	3.97	2.65	2.77
**Vitamins** (mg/100 g)	[13]						
Thiamin		0.383	0.413	0.385	0.763	0.316	0.646
Riboflavin		0.115	0.043	0.201	0.139	0.251	0.285
Niacin		5.464	4.308	3.627	0.961	4.270	4.604
Vitamin B6		0.300	0.509	0.622	0.119	0.294	0.318
Folate		0.038	0.020	0.019	0.056	0.038	0.019
Vitamin E		1.010	n.a.	0.490	n.a.	0.850	0.570
Vitamin K		0.019	n.a.	0.003	n.a.	0.060	0.022
**Phytochemicals ^§^** (mg/100 g)	[14,15,16]						
Phenolic acids		1.342	0.286	0.601	0.472	1.364	0.898
Ferulic acid		114	30	174	2	4	115
Flavonoids		36	n.a.	n.a.	7	7	15
Betaine		156	0.5	n.a.	55	n.a.	58
Carotenoids		0.33	0.04	1.45	0.03	n.a.	0.06
Alkylresorcinol		0.47	n.a.	n.a.	n.a.	1.89	0.75
Phytosterols		77.5	n.a.	14.8	n.a.	n.a.	102

n.a. = not available. **^§^** Mean values based on references.

**Table 2:** Randomized clinical trials on the effects of wholegrain on body weight.

Author (Reference)	Study Design	Study Population Participants Age BMI Health Status	Intervention and Doses	Duration Weeks	Observed Effects in Wholegrain Group
Pol et al., 2013 [38]	Meta-analyses	2060 M/F 18–70 years 18–35 kg/m^2^ -	wholegrain (mostly oat, wheat, barley, rye, rice: 18–136 g/day) vs. refined grain	2–16	= BW = WC ↓ Body fat
Kirwan et al., 2016 [39]	RCT, crossover	40 M/F 40 years 33 kg/m^2^ Healthy	wholegrain (wheat, rice, oat: 93 g/day) vs. refined grain	6	= BW =WC
Suhr et al., 2017 [40]	RCT, parallel	75 M/F 30–65 years 28 kg/m^2^ Healthy	*ad libitum* wholegrain rye-based foods (124 ± 12 g/day) vs. *ad libitum* wholegrain wheat-based foods (145 ± 12 g/day) vs. *ad libitum* refined wheat-based foods	6	Whole rye: ↓ BW = WC Whole wheat: = BW = WC
Li et al., 2016 [41]	RCT, parallel	287 M/F 59 years 27 kg/m^2^ T2DM	wholegrain oat-based foods (100 g/day) vs. wholegrain oat-based foods (50 g/day) vs. usual dietary habits	48	↓ BW

= no changes; ↓ significant decrease; BMI: body mass index; BW: body weight, T2DM: type 2 diabetes mellitus; F: female; M: male; WC: waist circumference; and RCT: randomized clinical trial.

**Table 3:** Randomized clinical trials on the effects of wholegrain on insulin resistance/sensitivity.

Author (Reference)	Study Design	Study Population Participants Age BMI Health Status	Intervention and Doses	Duration Weeks	Observed Effects in Wholegrain Group
Pereira et al., 2002 [50]	RCT cross-over	11 M/F 41.6 years 30.2 kg/m^2^ Hyperinsulinemic	wholegrain foods (mostly wheat, rice, rye, corn, oat, burley: 386 g/day) vs. refined grain	6	↑ IS (euglycemic hyperinsulinemic clamp tests)
Juntunen et al., 2003 [51]	RCT cross-over	20 F 59 years 28 kg/m^2^ Healthy	high fiber rye bread (208 g/day) vs. white wheat bread	8	= IS (FSIGT)
McIntosh et al., 2003 [52]	RCT cross-over	28 M 40–65 years 30 kg/m^2^ Healthy	wholegrain rye-based foods (230 g/day) vs. wholegrain wheat-based foods (230 g/day) vs. low fiber diet	4	= IS (HOMA)
Andersson et al., 2007 [53]	RCT cross-over	30 M/F 59 years 28.3 kg/m^2^ One criteria of MS	wholegrain foods (mostly wheat, oat, rye, rice: 112 g/day) vs. refined grain	6	= IS (euglycemic hyperinsulinemic clamp tests)
Katcher et al., 2008 [54]	RCT parallel	47 M/F 46 years 36 kg/m^2^ MS	wholegrain foods (mostly wheat, oat, rye, rice: 218 g/day) vs. refined grain	12	= IS (ISI during OGTT)
Giacco et al., 2010 [55]	RCT crossover	15 M/F 55 years 27 kg/m^2^ Healthy	wholegrain wheat-based foods (283 g/day) vs. refined grain	3	= IS (HOMA)
Brownlee et al., 2010 [56]	RCT parallel	216 M/F 46 years 30 kg/m^2^ Healthy	Wholegrain foods (wheat, oat, rice: 120 g/day) vs. wholegrain foods (wheat, oat, rice: 60 g/day) vs. refined grain	16	= IS (QUICKI)
Giacco et al., 2013 [57]	RCT parallel	133 M/F 40–65 years 31.4 kg/m^2^ MS	wholegrain foods (rye, wheat: 232 g/day) vs. refined grain	12	= IS (FSIGT)
Malin et al., 2018 [58]	RCT crossover	14 M/F 38 years 34 kg/m^2^ Healthy	wholegrain foods (wheat, oat, rice: 90 g/day) vs. refined grain	8	↑ IS (OGTT with isotopic tracer)
He et al., 2016 [59]	Meta-analyses	298 M/F 53 years 26 kg/m^2^ Overweight/T2DM	wholegrain oat-based foods (20–136 g/day) vs. refined grain foods	8	↑ IS (HOMA)

= no changes; ↑ significant increase; BMI: body mass index; T2DM: Type 2 Diabetes Mellitus; HOMA: Homeostatic model assessment; IS: insulin sensitivity; ISI: insulin sensitivity index; F: female. FSIGT: Frequently sampled intravenous glucose tolerance test; M: male; MS: Metabolic Syndrome; OGTT: oral glucose tolerance test; QUICKI: Quantitative insulin sensitivity check index; and RCT: randomized clinical trial.

**Table 4:** Randomized clinical trials on the effects of wholegrain on blood glucose regulation.

Author (Reference)	Study Design	Study Population Participants Age BMI Health Status	Intervention and Doses	Duration Weeks	Observed Effects in Wholegrain Group
**Fasting condition**					
Marventano et al., 2017 [60]	Meta-analyses	377 M/F 50 years 28 kg/m^2^ Healthy	Wholegrain foods (mostly wheat, rye, rice, barley, maize and oat) vs. refined grain	2–16	= glucose
**Postprandial condition**					
Marventano et al., 2017 [60]	Meta-analyses	377 M/F 50 years 28 kg/m^2^ Healthy	wholegrain foods (mostly rye, oat and barley) vs. refined grain foods	Acute meal studies	↓ glucose AUC
Lappi et al., 2013 [65]	RCT, Crossover	21 M/F 38–65 years 19–30 kg/m^2^ Healthy	wholegrain rye bread (180–300 g/day) vs. refined wheat bread	4	= glucose AUC
Giacco et al., 2014 [61]	RCT, Parallel	54 M/F 56 years 31.7 kg/m^2^ MS	wholegrain foods (wheat, oat, rye, barley: 268 g/day) vs. refined grain	12	= glucose AUC

= no changes; ↓ significant decrease; AUC: area under the curve; BMI: body mass index; F: female; M: male; MS: Metabolic Syndrome; and RCT: randomized clinical trial.

**Table 5:** Clinical trials on the effects of wholegrain on blood glucose, insulin, and HbA1c in patients with type 2 diabetes mellitus.

Author (Reference)	Study Design	Study Population Participants Age BMI Health Status	Intervention and Doses	Duration Weeks	Observed Effects in Wholegrain Group
**Fasting condition**					
Hou et al., 2015 [70]	Meta-analyses	306 M/F 60 years - T2DM	wholegrain oat-based foods (50–100 g/day) vs. refined grain foods	1–4	↓ glucose = insulin ↓ HbA1c = Insulin resistance (HOMA)
Shen et al., 2016 [71]	Meta-analyses	350 M/F 61 years 28 kg/m^2^ T2DM	wholegrain oat-based foods (2.5–5 g/day) vs. refined grain foods	3–8	↓ glucose = insulin ↓ HbA1c
Li et al., 2016 [41]	RCT, parallel	287 M/F 59 years 27 kg/m^2^ T2DM	wholegrain oat-based foods (100 g/day) vs. wholegrain oat-based foods (50 g/day) vs. usual dietary habits	48	↓ glucose ↓ insulin ↓ HbA1c
**Postprandial condition**					
Hou et al., 2015 [70]	Meta-analyses	306 M/F 60 years - T2DM	wholegrain oat-based foods (50–100 g/day) vs. refined grain foods	1–4	↓ AUC glucose
Li et al., 2016 [41]	RCT, parallel	287 M/F 59 years 27 kg/m^2^ T2DM	wholegrain oat-based foods (100 g/day) vs. wholegrain oat-based foods (50 g/day) vs. usual dietary habits	48	↓ glucose AUC

= no changes; ↓ significant decrease; AUC: area under the curve; BMI: body mass index; T2DM: Type 2 Diabetes Mellitus; F: female; HbA1c: glycosylated hemoglobin; M: male; and RCT: randomized controlled trial.

## Abbreviations

The following abbreviations are used in this manuscript:

T2DM	Type 2 Diabetes Mellitus
HbA1c	Glycated Hemoglobin A1c
BMI	Body Mass Index
LDL	Low Density Lipoprotein
HDL	High Density Lipoprotein
HOMA	Homeostatic Model Assessment
